# Obeticholic Acid and Edaravone Protect Against Cisplatin‐Induced Hepatotoxicity Through Modulation of Keap1/Nrf2/ARE, TNF‐α/NF‐κB, and AKT/GSK‐3β Pathways

**DOI:** 10.1002/jbt.71101

**Published:** 2026-09-03

**Authors:** Fares E. M. Ali, Adel G. Bakr, Abdel‐Gawad S. Shalkami, Ehab A. M. El‐Shoura, Lamiaa Khalaf Ahmed, Ali F. Abd‐Elsalam, Ahmed A. N. Ahmed, Emad H. M. Hassanein

**Affiliations:** ^1^ Department of Pharmacology and Toxicology, Faculty of Pharmacy Al‐Azhar University Assiut Egypt; ^2^ Michael Sayegh, Faculty of Pharmacy Aqaba University of Technology Aqaba Jordan; ^3^ Department of Clinical Pharmacy, Al‐Rayan National College of Health Sciences Al‐Rayan National College Medina Saudi Arabia; ^4^ Department of Clinical Pharmacy, Faculty of Pharmacy Al‐Azhar University Assiut Egypt; ^5^ Biochemistry and Molecular Biology Department, Faculty of Pharmacy (Girls) Al‐Azhar University Cairo Egypt; ^6^ Department of Pharmacology, Faculty of Medicine Al‐Azhar University Assiut Egypt

**Keywords:** AKT/GSK‐3β, cisplatin, edaravone, Keap1/Nrf2/ARE, obeticholic acid

## Abstract

Hepatotoxicity is one of the most crucial side effects of chemotherapy administration. Obeticholic acid (OCA) is a semisynthetic bile acid and farnesoid X receptor (FXR) agonist derived from chenodeoxycholic acid, with reported antioxidant and anti‐inflammatory effects in liver disorders. This study investigated the hepatoprotective effect of OCA against commonly used chemotherapy cisplatin (CP)‐induced hepatotoxicity in rats, as well as the modulatory effects of edaravone (EDA), a potent free radical scavenger, on its effects. Rats were divided into five groups: control (received vehicle), CP (7.5 mg/kg), EDA (30 mg/kg) + CP, OCA (30 mg/kg) + CP, and EDA + OCA + CP. The results of the present study demonstrated that both OCA and EDA significantly mitigated liver damage caused by CP, as evidenced by restoring liver enzymes and histological structure, reestablishment of oxidant/antioxidant status, suppression of inflammation, and attenuation of pro‐death signaling. The study highlights the role of key molecular pathways, including Keap1/Nrf2/HO‐1,HO‐1, TNF‐α/NF‐κB, and AKT/GSK‐3β, in the hepatoprotective mechanisms of OCA. Collectively, these findings suggest that OCA and EDA, particularly in combination, attenuate CP‐induced hepatotoxicity and are associated with coordinated modulation of oxidative stress, inflammatory signaling, and AKT/GSK‐3β‐associated pro‐survival/pro‐death pathways.

## Graphical abstract

The graphical abstract explores the possible underlying molecular mechanisms of Obeticholic acid against hepatotoxicity induced by chemotherapy exposure.

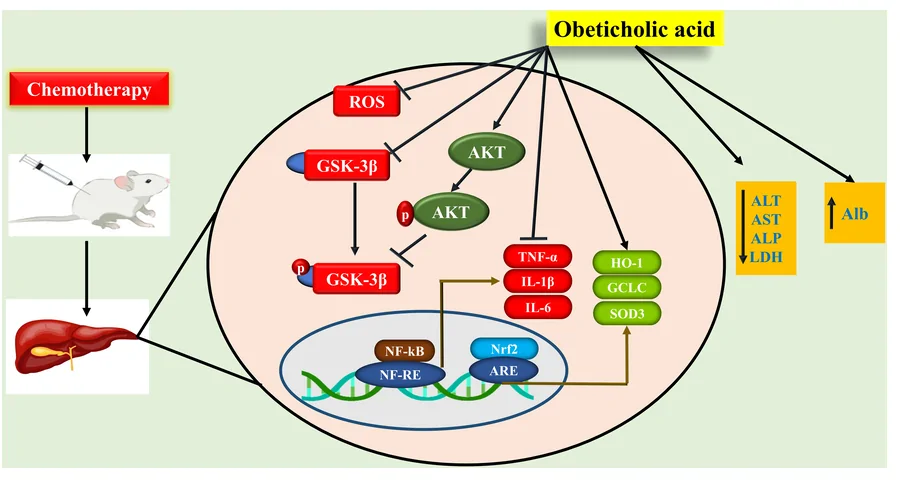

## 1 Introduction

Drug‐induced liver injury (DILI) remains the leading cause of liver failure. Chemotherapy is one of the main drug categories most often linked to DILI, and it is a significant cause of illness and death. Therefore, patients undergoing chemotherapy need a careful assessment of liver function before treatment [1, 2]. Cisplatin (CP) has gained significant clinical usage in treating several malignancies due to its remarkable tissue penetration and broad‐spectrum anticancer properties [3]. Nonetheless, the clinical application of CP is hindered by severe adverse effects such as nephrotoxicity and hepatotoxicity [4]. Multiple investigations have indicated that the prolonged accumulation of high CP doses can induce hepatotoxicity through oxidative injury, inflammatory responses, and hepatocellular apoptosis [5, 6].

Even while hepatotoxicity and its underlying mechanism and the investigation of preventive pharmacological therapies have received relatively less attention, CP accumulation in the liver is notable for being second only to that in the kidney [7]. CP‐induced hepatotoxicity is prominently caused by oxidative stress, which arises from the detrimental impact of reactive oxygen species (ROS) [8]. Moreover, the intracellular buildup of ROS is closely associated with the activation of pro‐inflammatory cytokines through the nuclear factor kappa B (NF‐ĸB) pathway and various pro‐apoptotic cellular signaling pathways [9].

Prior investigations have provided evidence that CP administration leads to enhanced expression of pro‐inflammatory cytokines, including tumor necrosis factor‐alpha (TNF‐α), interleukin‐1 beta (IL‐1β), and interleukin‐6 (IL‐6) [10]. On the other hand, the nuclear factor erythroid 2‐related factor 2 pathway (Nrf2) signaling pathway is a vital cellular defense mechanism against oxidative stress and inflammation. Nrf2 is a transcription factor that regulates antioxidant response and detoxification genes. Activation of Nrf2 occurs in response to oxidative stress, electrophilic compounds, and pro‐inflammatory signals. In normal conditions, Nrf2 is bound to Keap1. However, under oxidative stress or electrophilic insults, Nrf2 is released from Keap1 and translocates into the nucleus [11]. Activation of Nrf2 results in the upregulation of many antioxidant enzymes and cytoprotective proteins such as heme oxygenase 1 (HO‐1), superoxide dismutase‐3 (SOD3), and glutamate‐cysteine ligase catalytic subunit (GCLC). These proteins combat reactive oxygen species, enhance cellular antioxidant capacity, and maintain redox homeostasis [12, 13].

Obeticholic acid (OCA) is a derivative of 6α‐ethyl chenodeoxycholic acid (CDCA) that functions as an agonist of the farnesoid X receptor (FXR) for the treatment of liver disorders [14, 15, 16]. OCA significantly improves liver function tests among patients with primary biliary cirrhosis [17], non‐alcoholic steatohepatitis [18], and fatty liver disease [19]. Moreover, a substantial body of animal studies has accumulated, indicating the effective anti‐inflammatory properties of OCA [20, 21]. Pretreatment with OCA has been shown to inhibit hepatocyte apoptosis induced by carbon tetrachloride (CCL4) by suppressing the NF‐κB‐mediated inflammatory response [22]. Edaravone is an innovative free radical scavenger proposed to treat many diseases [23, 24]. It effectively inhibits the generation of ROS and suppresses inflammation in different models [25, 26, 27]. The primary objective of our study was to investigate the impact of the Keap1/Nrf2/ARE, TNF‐α/NF‐κB, and AKT/GSK‐3β pathways on chemotherapy‐induced hepatotoxicity, as well as the hepatoprotective effect of OCA against CP ‐induced hepatotoxicity in rats. Additionally, we examined the modulatory effects of EDA, a potent free radical scavenger, and the underlying mechanisms.

## 2 Materials and Methods

### 2.1 Drugs

Cisplatin MYLAN® vial (1 mg/1 mL) was used in the current work. ED (CAS No. 89‐25‐8) and OCA (CAS No. 459789‐99‐2) were purchased from Sigma Aldrich (St. Louis, MO, USA).

### 2.2 Animals

In our study, 40 adult male Wistar rats, weighing between 180 and 200 g, were an average of 12–14 weeks old. Rats were purchased from Assiut University's Central Animal House (Assiut, Egypt). The rats were kept in a 12‐h cycle of light and darkness. The animals were housed in plastic cages (four rats/cage), fed with standard feeding pellets, and provided with tap water *ad libitum*. The present animal procedure was approved by the Faculty of Pharmacy ethical committee at Al‐Azhar University (Approval number: ZA‐AS/PH/6/C/2022) and followed the ARRIVE Guidelines for reporting in vivo experiments.

### 2.3 Experimental Design

Rats were randomly allocated into five groups and treated as follows (*n* = 8/group):

**Group 1:** Served as a normal control group; rats received vehicles (0.5% carboxymethyl cellulose; CMC, 1 ml/rat) for 10 days. **Group 2:** Served as the disease control group; rats received a single intraperitoneal dose of CP (7.5 mg/kg) on day 5 of the experimental design [28]. **Group 3:** Rats received intraperitoneal EDA (30 mg/kg/day) for 10 days, besides CP (7.5 mg/kg, i.p.) on day 5 of the design [29]. **Group 4:** Rats received OCA (30 mg/kg, p.o.) for 10 days, besides CP (7.5 mg/kg/day, i.p.) on day 5 of the design [30, 31]. **Group 5:** Rats received OCA and EDA for 10 days, besides CP, at doses mentioned before on day 5 of the design. The selection of doses was based on our previous preliminary data as well as previous reports.

### 2.4 Sampling Collection

After 6 h from the last dose, all rats were anesthetized with ketamine (50 mg/kg, i.p.). Blood was obtained from the retroorbital plexus and allowed to clot for serum preparations. The serum was separated by centrifugation at 4000 rpm for 10 min for the assessment of alanine transaminase (ALT), aspartate aminotransferase (AST), alkaline phosphatase (ALP), lactate dehydrogenase (LDH), and albumin. Then, the rats were sacrificed, and their livers were dissected and washed with ice‐cold saline. A small portion of hepatic tissue from each group was preserved in 10% neutral formalin for histopathological examination and immunohistochemical assessment of Nrf2, HO‐1, and NF‐κB. A large part of the hepatic tissues was divided into two parts. One section was homogenized in 0.1 M phosphate buffer at pH 7.4 to produce 10% tissue homogenates for the determination of reduced glutathione (GSH), superoxide dismutase (SOD), and malondialdehyde (MDA), and for ELISA assessments. The other section was handled using RNAlater® for quantitative real‐time PCR analysis of Keap1, Nrf2, GCLC, SOD3, and HO‐1 gene expression. The last portion was kept in lysis buffer for western blotting analyses for protein kinase B (AKT), p‐AKT, glycogen synthase kinase‐3β (GSK‐3β), and p‐GSK‐3β.

### 2.5 Liver Function Assessment

The levels of liver enzymes, including ALT (CAT# 1001170), AST (CAT# 1001160), ALP (CAT# 41240), LDH (CAT# 1001260), and albumin (CAT# 1001020), were assessed based on the manufacturer's directions (SPINREACT, Spain).

### 2.6 Estimation of Antioxidant and Oxidant Parameters

To examine the oxidative stress, the enzymatic activity of SOD, and the contents of lipid peroxidation, MDA, and GSH in the homogenates of liver tissues were measured by the method documented earlier by Marklund [32], Mihara, Uchiyama [33], and Sedlak, Lindsay [34], respectively.

### 2.7 Determination of Inflammatory Mediators

The hepatic TNF‐α (CAT# E‐EL‐R2856), IL‐1β (CAT# E‐EL‐R0012), and IL‐6 (CAT# E‐EL‐R0015) levels were measured by the ELISA technique based on the manufacturer's directions and the basic principle of Van Weemen, Schuurs [35]. The kits were purchased from ELABSCIENCE (Wuhan, China).

### 2.8 Gene Expression Assessment

mRNA expression of Keap1, Nrf2, GCLC, SOD3, and HO‐1 genes was quantified by quantitative real‐time PCR. Briefly, total RNA was separated by TRIzol reagent (CAT# 15596026, Invitrogen, USA). The obtained RNA quantity and purity were determined by nanodrop and then reverse transcribed into cDNA using RevertAid First Strand cDNA Synthesis Kit (CAT# K1622, ThermoFisher Scientific, USA). The resultant cDNA was amplified via Universal SYBR Green Master Mix (CAT# 4309155, Applied Biosystem, USA). The data obtained were evaluated using the 2^−ΔΔCt^ equation [36] and referenced GAPDH as a housekeeping gene. The primers are indexed in Table 1.

**TABLE 1:** Quantitative real‐time PCR primers.

Gene	Forward primer	Reverse primer
Keap1	TCAGCTAGAGGCGTACTGGA	TTCGGTTACCATCCTGCGAG
Nrf2	ATTGCTGTCCATCTCTGTCAG	GCTATTTTCCATTCCCGAGTTAC
HO‐1	TGCTTGTTTCGCTCTATCTCC	CTTTCAGAAGGGTCAGGTGTC
GCLC	GTTGTTACTGAATGGCGGCG	CGGCGTTTCCTCATGTTGTC
SOD3	TCCTGGCTCACAGAACGAATG	AGGTCTTTGGAGTGCGTGTC
GAPDH	TGCTGGTGCTGAGTATGTCG	TTGAGAGCAATGCCAGCC

### 2.9 Western Blotting

A small section of liver tissue was lysed using RIPA buffer, including a protease inhibitor cocktail. Total proteins were estimated from the resultant supernatant based on the method described by Bradford [37]. The proteins were loaded (50 µg in each lane) and then electrophoresed on SDS‐polyacrylamide gels and relocated to a polyvinylidene difluoride membrane (Immobilon®‐P PVDF Membrane, CAT# IPVH00010, Merk, Germany) through a semi‐dry transfer manner [38]. After being blocked with 5% non‐fat dried milk powder for 2 h, the membrane was mixed with primary antibodies of anti‐GSK‐3β (CAT# sc‐377213, Sant Cruz, USA), anti‐p‐GSK‐3β (CAT# sc‐373800, Sant Cruz, USA), anti‐AKT (CAT# E‐AB‐15441, ELABSCIENCE, China), anti‐p‐AKT (CAT# E‐AB‐20804, ELABSCIENCE, China), and Anti‐β‐actin (CAT# E‐AB‐20031, ELABSCIENCE, China) overnight at 4°C. Then, the membranes were handled with secondary antibodies at 37°C for 1 h. The bands were visualized using the BCIP/NBT system (CAT# BWR1067, Biospes, China). The intensity of the bands was analyzed using ImageJ® software, and the values of each band were represented as the relative protein expression referenced to β‐actin bands.

### 2.10 Histological Examinations

Liver sections were settled in 10% neutral formalin and then desiccated, cleared, fixed in paraffin, sliced off (4–5 µm), and stained with periodic acid‐Schiff (PAS) solutions [39] and hematoxylin and eosin (H&E), based on instructions notarized by Feldman, Wolfe [40]. The slides were evaluated randomly using a light microscope (Olympus, USA).

### 2.11 Immunohistochemical Studies

Liver slices were used for the immunohistochemical staining of Nrf2, HO‐1, and NF‐κB determination. Briefly, portions were mixed with primary anti‐Nrf2 (CAT# YPA1865, Biospes, China), anti‐HO‐1 (CAT# YPA1919, Biospes, China), and anti‐NF‐κB (CAT# abx012874, Abbexa, UK) antibodies. Then, the tissues were incubated with matched secondary antibodies for 1 h. The antigen‐antibody reactions evolved with 0.01% H_2_O_2_ and 0.05% diaminobenzidine for about 3 min. The slides were counterstained with hematoxylin. A light microscope (Olympus, USA) with a digital camera was used to take the photomicrographs. Using the ImageJ® software, the area percentage of immunoreactivity was measured.

### 2.12 Statistical Analysis

Multiple statistical comparisons were done via one‐way ANOVA, followed by Tukey's post‐hoc test. Data are introduced by Mean ± standard error of the mean. The level of significance at *p‐*value < 0.05 was considered statistically significant. Group Differences were made through GraphPad Prism software (Version 8.0, USA).

## 3 Results

### 3.1 OCA Ameliorates Chemotherapy‐Induced Hepatic Injury

**FIGURE 1:**
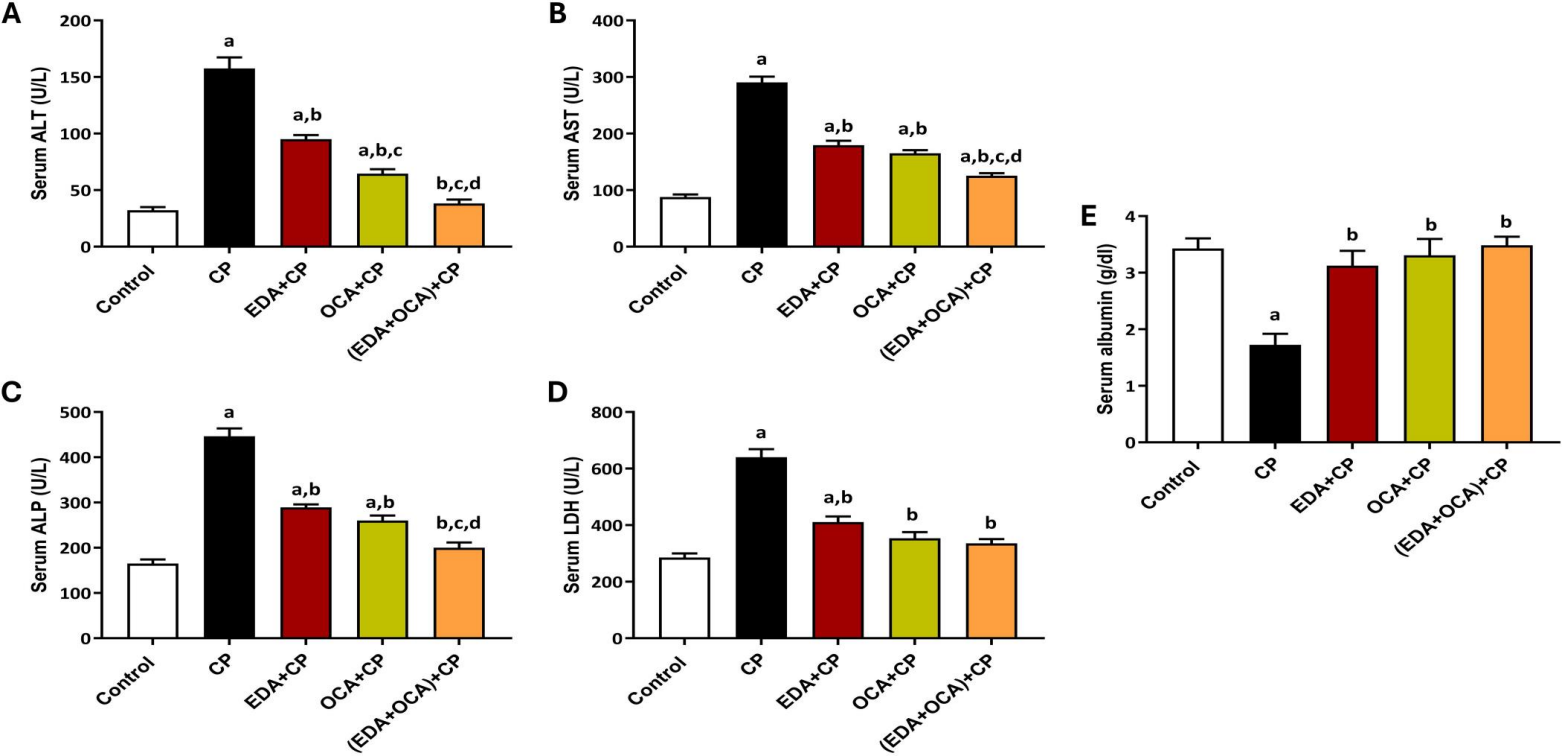
Effect of OCA, EDA, and their combination treatment on CP‐induced hepatic toxicity. Colorimetric investigation of ALT (A), AST (B), ALP (C), LDH (D), and albumin (E) was conducted. Data are presented as mean ± SEM of six different values. A one‐way ANOVA test was conducted for statistical analysis. a: indicated a significant difference from the normal control group at *p*‐value < 0.05. b: indicated a significant difference from the CP control group at *p*‐value < 0.05. c: indicated a significant difference from the EDA + CP group at *p*‐value < 0.05. d: indicated a significant difference from the OCA + CP group at *p*‐value < 0.05.

Initially, the administration of CP led to a notable increase in the concentrations of liver function enzymes, including ALT, AST, ALP, and LDH. At the same time, the albumin levels exhibited a decrease in rats treated with CP. On the contrary, the administration of OCA effectively counteracted these effects, significantly reducing ALT, AST, ALP, and LDH levels and increasing albumin levels. Notably, the combination of OCA plus EDA exhibited a higher protective effect on liver function biomarkers than that of OCA alone (Figure 1).

### 3.2 OCA Mitigates Chemotherapy‐Induced Hepatic Histopathological Changes

**FIGURE 2:**
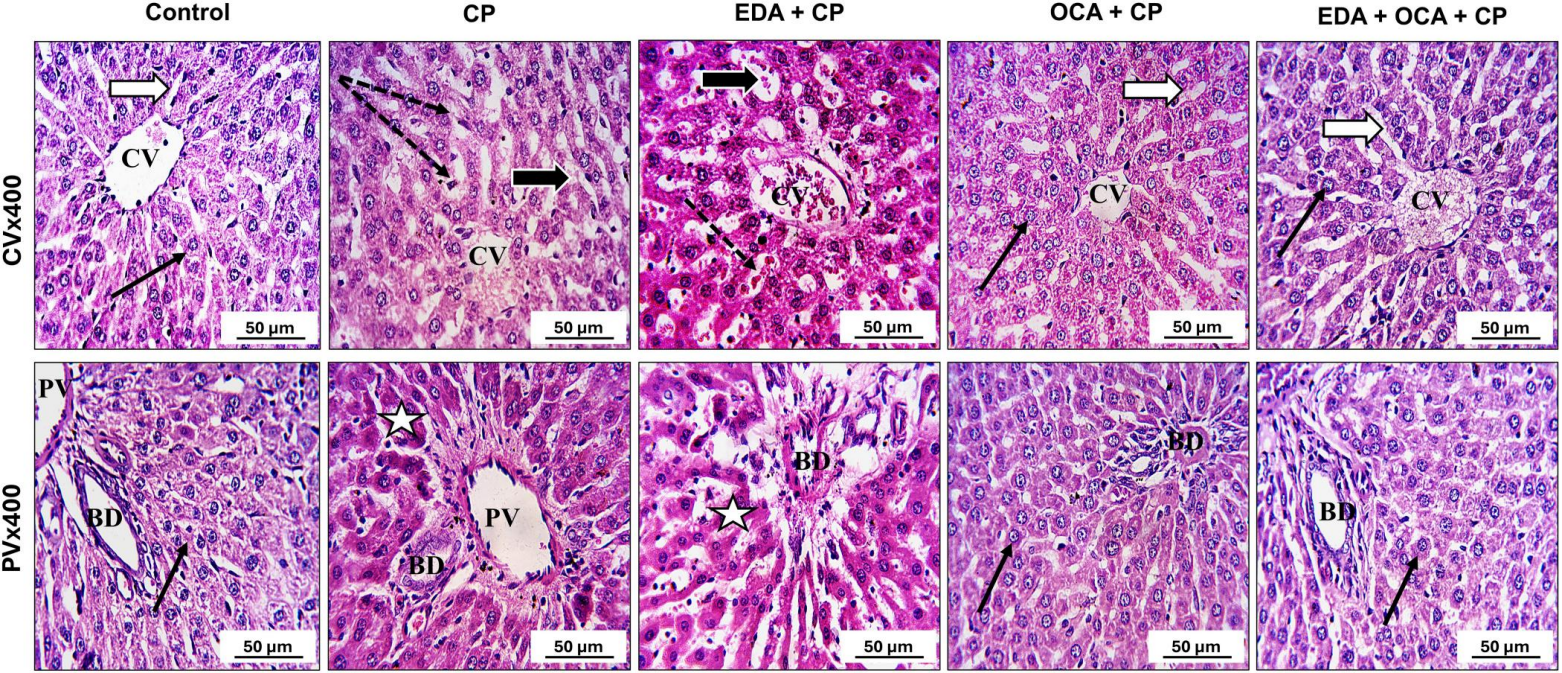
Representative photomicrographs of H&E‐stained liver sections from the different experimental groups: (A) Control, (B) CP, (C) EDA + CP, (D) OCA + CP, and (E) OCA + EDA + CP. The control group showed normal hepatic architecture with preserved hepatocytes and narrow sinusoids. The CP group showed marked histopathological alterations, including shrunken hepatocytes, darkly stained cytoplasm, sinusoidal dilatation, prominent Kupffer cells, and degenerative changes in the central/portal regions. The EDA + CP group showed partial improvement, whereas the OCA + CP group showed marked protection. The OCA + EDA + CP group demonstrated the greatest preservation of hepatic architecture, with sections nearly comparable to those of the control group. Images were captured at the same magnification. Scale bar = 50 μm.

Histological examination using H&E stain for general structure showed that normal control rats displayed normal hepatocytes and had rounded active nuclei separated by narrow hepatic blood sinusoids lined by endothelial cells without prominence of Kupffer phagocytic cells. Rats receiving a single CP dose showed shrunken hepatocytes with darker‐stained cytoplasm and smaller nuclei. Blood sinusoids between cell cords are dilated with evident prominence of granulate Kupffer cells (dotted arrows). The central vein endothelial lining looked degenerated and ill‐defined. Hepatocytes near the portal vein showed dark, deformed morphology consistent with marked cellular injury. In the OCA + CP‐treated group, marked protection was observed, and hepatic tissue looked healthy. In the EDA + CP‐treated group, mild protection was observed, as well as sinusoidal dilatation with prominence of Kupffer cells, but with less frequency than in the CP non‐treated group. Interestingly, combinations of EDA + OCA showed more protection observed with hepatic tissue that looked more or less similar to normal control (Figure 2).

### 3.3 OCA Counteracts Chemotherapy‐Induced Hepatic Oxidative Stress

**FIGURE 3:**
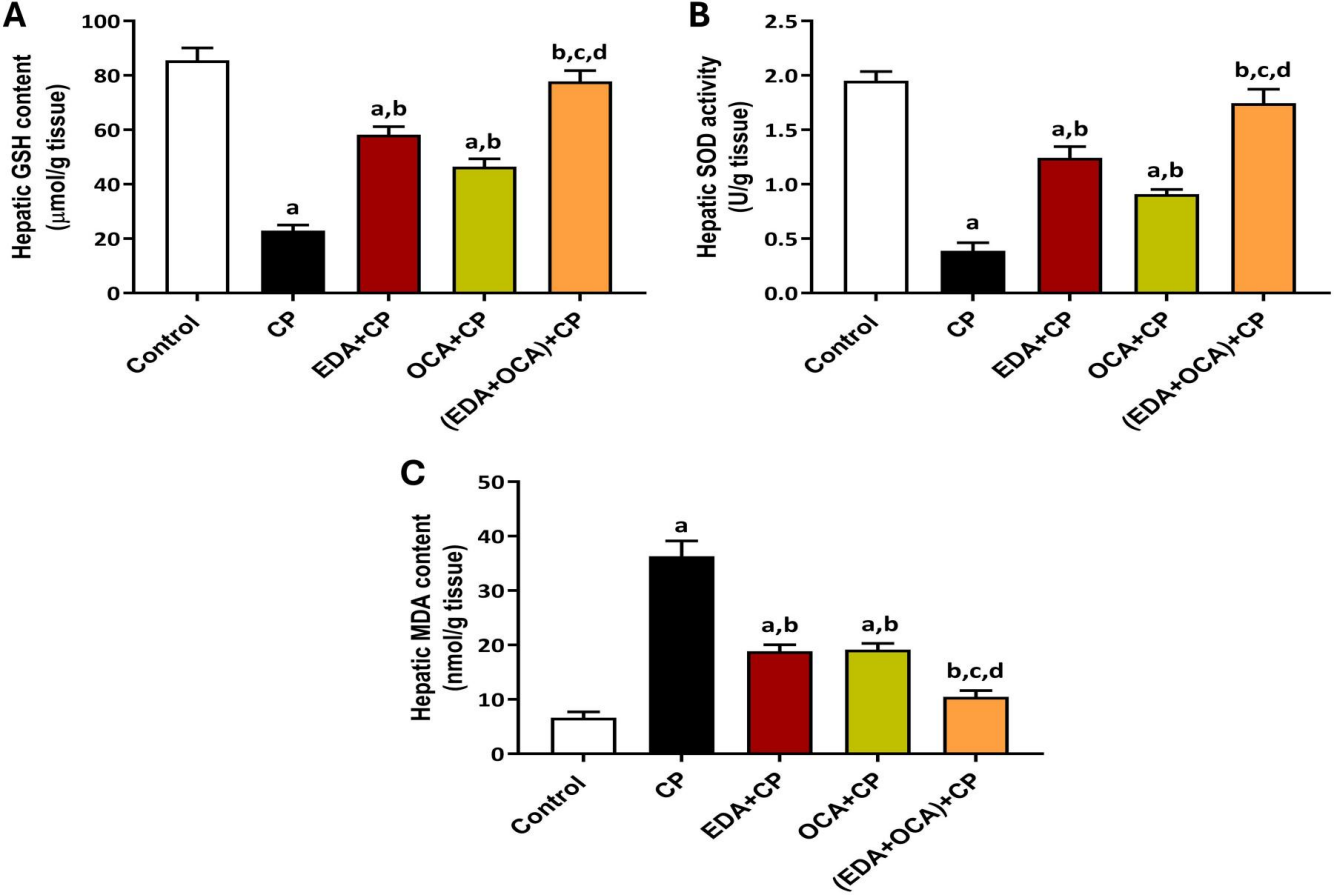
Effect of OCA, EDA, and their combination on cellular hepatic oxidative stress induced by CP. Colorimetric determination of GSH (A), SOD (B), and MDA (C) was conducted. Data are presented as mean ± SEM of six different values. A one‐way ANOVA test was conducted for statistical analysis. a: indicated a significant difference from the normal control group at *p*‐value < 0.05. b: indicated a significant difference from the CP control group at *p*‐value < 0.05. c: indicated a significant difference from the EDA + CP group at *p*‐value < 0.05. d: indicated a significant difference from the OCA + CP group at *p*‐value < 0.05.

Evidently, the group treated with CP exhibited decreased levels of GSH and SOD and an elevation of lipid peroxidation biomarker MDA in comparison to the control group. Conversely, co‐treatment with OCA effectively restored the levels of antioxidant enzymes while significantly reducing MDA levels. Compared to OCA alone, combination therapy with EDA showed a significant antioxidant effect, as shown in Figure 3.

**FIGURE 4:**
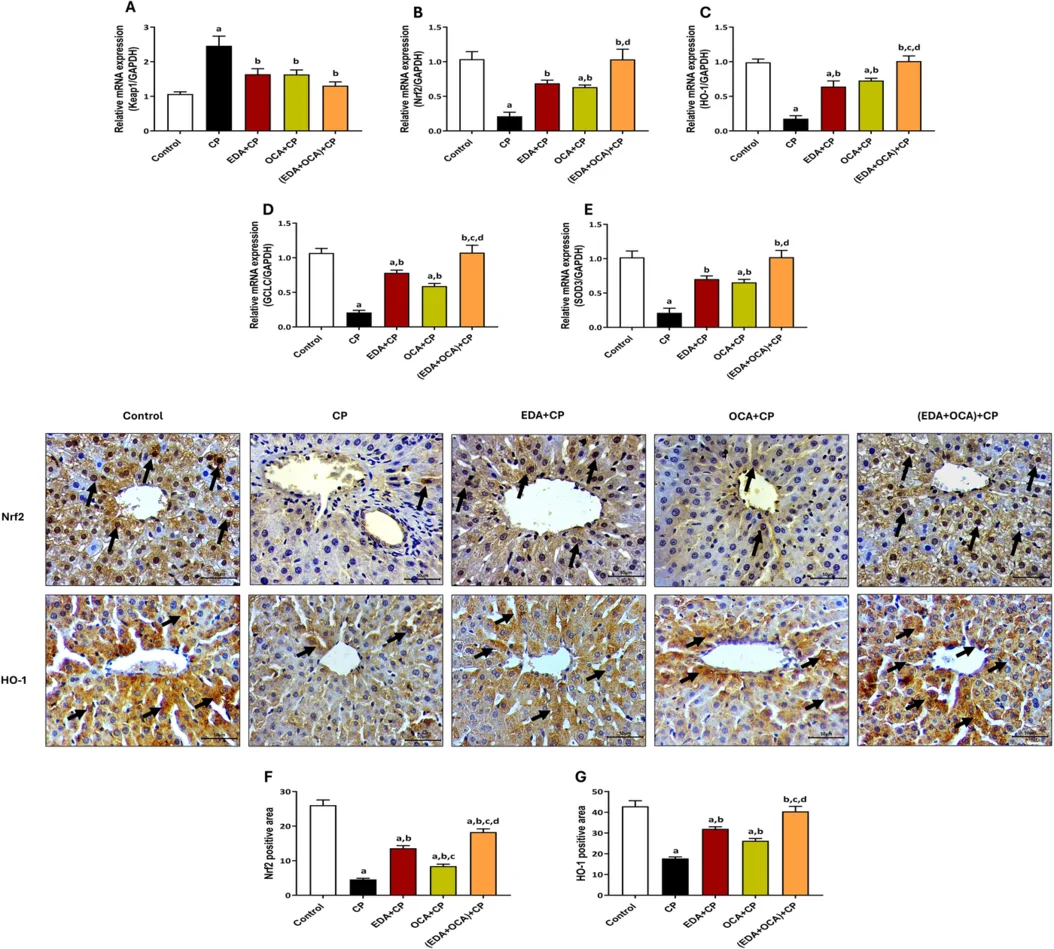
Effect of OCA, EDA, and their combination on hepatic mRNA expression of Keap1 (A), Nrf2 (B), HO‐1 (C), GCLC (D), and SOD3 (E). qRT‐PCR was conducted. Protein expression of Nrf2 and HO‐1 was conducted using immunohistochemistry analysis. (F) Bar graphs represent quantitative analysis of Nrf2 protein expression. (G) Bar graphs represent quantitative analysis of HO‐1 protein expression. Data are presented as mean ± SEM (n = 4). A one‐way ANOVA test was conducted for statistical analysis. a: indicated a significant difference from the normal control group at *p*‐value < 0.05. b: indicated a significant difference from the CP control group at *p*‐value < 0.05. c: indicated a significant difference from the EDA + CP group at *p*‐value < 0.05. d: indicated a significant difference from the OCA + CP group at *p*‐value < 0.05. Scale bar = 50 μm.

Moreover, our immunohistochemical analysis demonstrated a significant downregulation of Nrf2 and HO‐1 in the CP‐treated group. However, noteworthy upregulation was observed in the groups treated with OCA, EDA, and their combination, as evidenced by a substantial restoration of Nrf2 and HO‐1 immunopositivity, as depicted in Figure 4. Furthermore, CP significantly upregulated Keap‐1 mRNA expression, while significantly downregulating Nrf2, GCLC, SOD3, and HO‐1, as shown in Figure 4. In contrast, pretreatment with OCA counteracted these alterations by reducing Keap‐1 expression and restoring Nrf2, GCLC, SOD3, and HO‐1 expression. Collectively, treatment with the OCA + EDA combination produced a more pronounced normalization of Keap‐1 and greater restoration of Nrf2‐associated antioxidant gene expression compared with OCA alone.

### 3.4 OCA Dampens Chemotherapy‐Induced Hepatic Inflammation

**FIGURE 5:**
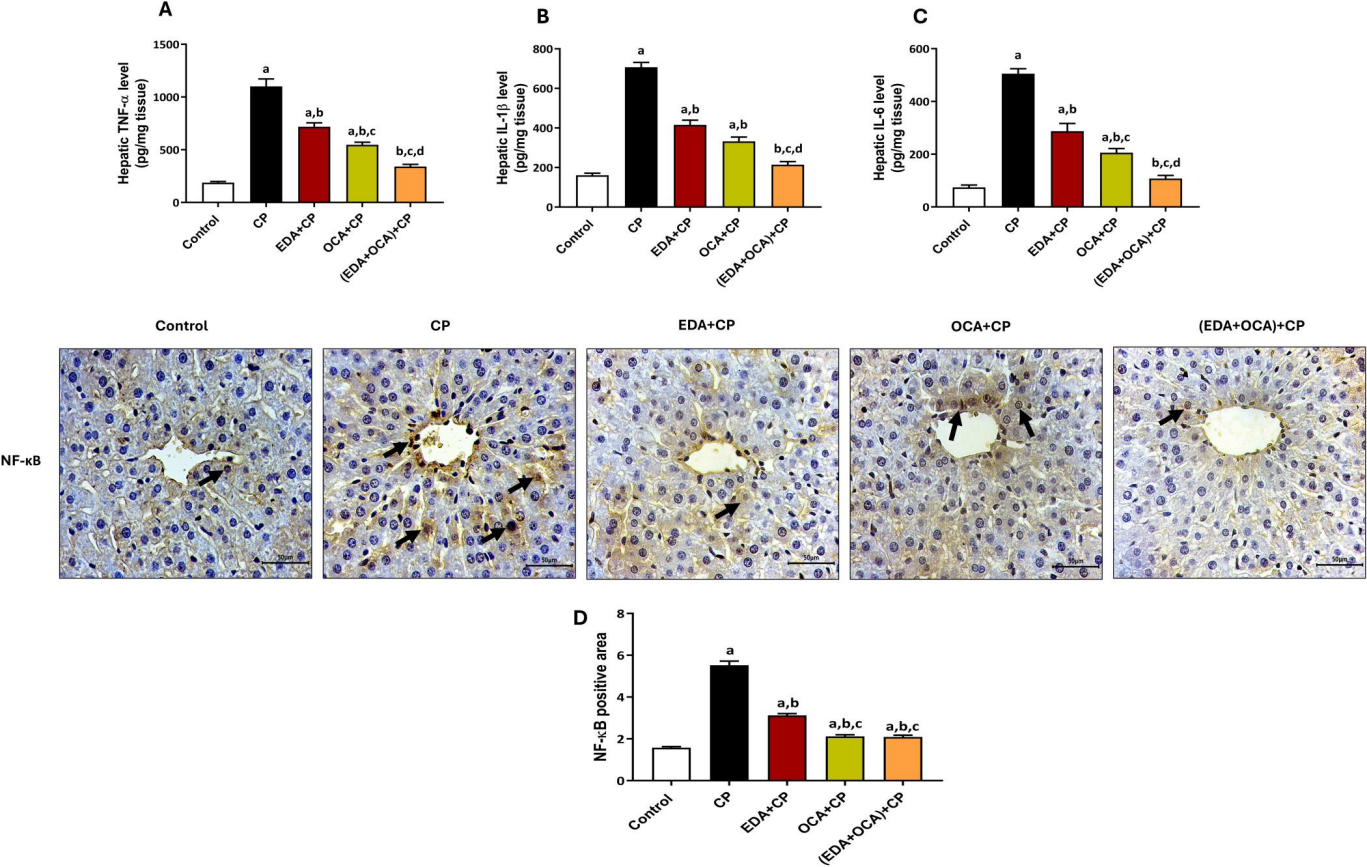
Effect of OCA, EDA, and their combination on hepatic inflammatory response induced by CP. Hepatic levels of TNF‐α (A), IL‐1β (B), and IL‐6 (C) were assessed using the ELISA technique. Protein expression of NF‐κB was conducted using immunohistochemistry analysis. (D) Bar graphs represent quantitative analysis of NF‐κB protein expression. Data are presented as mean ± SEM (*n* = 6). A one‐way ANOVA test was conducted for statistical analysis. a: indicated a significant difference from the normal control group at *p*‐value < 0.05. b: indicated a significant difference from the CP control group at *p*‐value < 0.05. c: indicated a significant difference from the EDA + CP group at *p*‐value < 0.05. d: indicated a significant difference from the OCA + CP group at *p*‐value < 0.05. Scale bar = 50 μm.

Injection of CP resulted in a significant upregulation of TNF‐α, IL‐1β, IL‐6, and NF‐κB expression in the hepatic tissue as compared to the normal control. Conversely, rats treated with OCA exhibited a significant decrease in TNF‐α, IL‐1β, IL‐6, and NF‐κB expressions as compared to the CP control group, as illustrated in Figure 5. Interestingly, the combination therapy of OCA plus EDA showed a more pronounced anti‐inflammatory effect than either treatment alone.

### 3.5 Effect of OCA, EDA, and Their Combination on Hepatic AKT/GSK‐3β Signal

**FIGURE 6:**
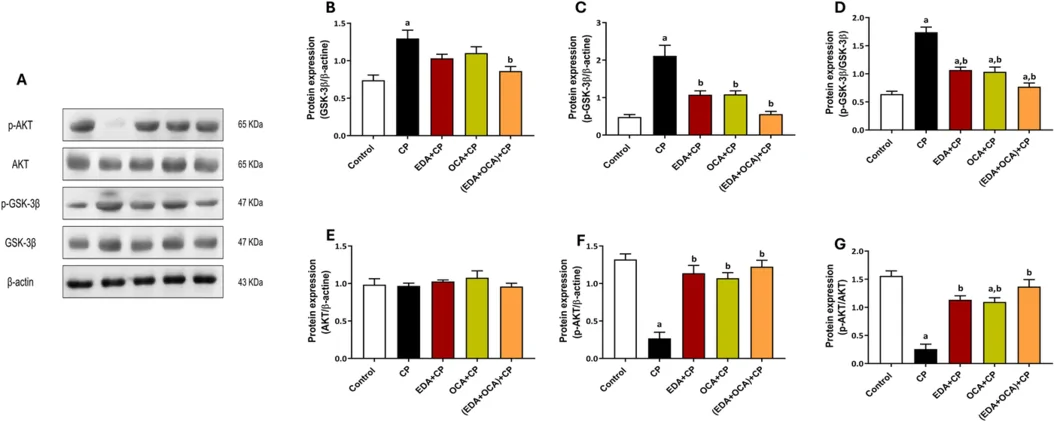
Effect of OCA, EDA, and their combination on hepatic AKT/GSK‐3β signal. (A) Western blot bands of GSK‐3β, p‐GSK‐3β, AKT, p‐AKT, and β‐actin protein expression. (B) Bar graphs represent the semiquantitative analysis of GSK‐3β protein expression. (C) Bar graphs represent the semiquantitative analysis of p‐GSK‐3β protein expression. (D) Bar graphs represent the semiquantitative analysis of p‐GSK‐3β/GSK‐3β protein expression. (E) Bar graphs represent the semiquantitative analysis of AKT protein expression. (F) Bar graphs represent the semiquantitative analysis of p‐AKT protein expression. G) Bar graphs represent the semiquantitative p‐AKT/AKT protein expression. Data are presented as mean ± SEM with three different values. A one‐way ANOVA test was conducted for statistical analysis. a: indicated a significant difference from the normal control group at *p*‐value < 0.05. b: indicated a significant difference from the CP control group at *p*‐value < 0.05. c: indicated a significant difference from the EDA + CP group at *p*‐value < 0.05. d: indicated a significant difference from the OCA + CP group at *p*‐value < 0.05.

Furthermore, western blot analysis demonstrated that CP significantly increased total GSK‐3β and p‐GSK‐3β protein expression, while reducing p‐AKT expression and the p‐AKT/AKT ratio. Treatment with OCA, EDA, and especially their combination attenuated these alterations by restoring AKT activation and reducing total GSK‐3β abundance. Because p‐GSK‐3β also declined in parallel with total GSK‐3β, this finding was interpreted as an overall reduction in GSK‐3β‐associated signaling rather than an isolated change in phosphorylation status (Figure 6).

## 4 Discussion

The main goal of our study was to examine the effects of the Keap1/Nrf2/ARE, TNF‐α/NF‐κB, and AKT/GSK‐3β pathways on chemotherapy‐induced hepatotoxicity, as well as the protective role of OCA against CP‐induced hepatotoxicity in rats. We also explored the modulatory effects of EDA, a powerful free radical scavenger, and the mechanisms behind these effects. Our study shows that OCA significantly reduced CP‐induced liver damage, restored liver enzyme levels and histology, improved the oxidant/antioxidant balance, reduced inflammation, and modulated hepatocellular stress/survival‐related signaling. It highlights key pathways, such as Keap1/Nrf2/HO‐1, TNF‐α/NF‐κB, and AKT/GSK‐3β, involved in OCA's liver protection. OCA combined with EDA offers greater protection than OCA alone.

Herein, we evaluated liver integrity by measuring ALT, AST, ALP, and LDH levels. Our findings revealed that CP administration increased ALT, AST, ALP, and LDH serum levels, indicating hepatocyte and cholangiocyte damage and subsequent liver dysfunction [41]. Additionally, albumin levels, which reflect liver synthetic and secretory functions, were assessed. Injured liver cells lose their capacity to synthesize albumin and eliminate bilirubin, leading to alterations in their serum levels [42]. In our investigation, CP injection resulted in a significant decrease in albumin levels. Conversely, our results demonstrated that administering OCA improved liver function biomarkers. Moreover, the hepatoprotective effects of OCA were simultaneously validated through the mitigation of CP‐induced histopathological alterations. In this regard, the ability of OCA to restore liver function and cellular integrity could be attributed to the antioxidant and anti‐inflammatory properties of both drugs, which may contribute to reduced cellular injury and decreased leakage of liver enzymes into the bloodstream.

Cisplatin administration induces oxidative stress in the liver, leading to an imbalance between the production of ROS and antioxidant defense mechanisms. This results in cellular damage and impaired liver function. Additionally, CP triggers an inflammatory response by releasing pro‐inflammatory cytokines and recruiting immune cells, exacerbating tissue damage and liver injury [43]. The interplay of oxidative stress and inflammation intensifies CP‐induced liver toxicity, contributing to the overall liver damage observed during CP therapy [43]. Our results revealed that CP increased the production of the lipid peroxidation marker, MDA, and depleted all the antioxidants, including GSH and SOD. Interestingly, GSH and SOD play vital roles in cellular defense against oxidative stress [44, 45, 46, 47]. Additionally, MDA is a reliable biomarker for oxidative damage, specifically lipid peroxidation induced by ROS. Elevated MDA levels effectively indicate heightened oxidative stress and lipid impairment, providing a valuable indicator of cellular oxidative damage [48]. On the other hand, a significant improvement in the oxidative status was seen after treatment with OCA and its combination with EDA, as they restored all the antioxidants and reduced the production of MDA. Results are consistent with earlier findings [49, 50, 51]. OCA and EDA, through their ROS‐scavenging and antioxidant‐boosting properties, counteract these effects, highlighting their potential as protective agents against cisplatin‐induced liver damage.

Nrf2 is a transcription factor responsible for regulating the expression of genes related to antioxidant defense [52, 53]. GCLC, an enzyme, is vital in synthesizing GSH, a key antioxidant in cells [54]. Nrf2, when translocated to the nucleus, activates the expression of various genes that contribute to cellular defense mechanisms. HO‐1, an enzyme induced by Nrf2, participates in heme breakdown and possesses potent dual antioxidant and anti‐inflammatory effects [52], while SOD3, an antioxidant enzyme, helps scavenge superoxide radicals [55]. Mechanistically, we found that CP increased Keap‐1 mRNA expression while decreasing Nrf2 and its downstream antioxidant targets, including GCLC, SOD3, and HO‐1, indicating suppression of Nrf2 signaling. In contrast, OCA and EDA, particularly in combination, attenuated the CP‐induced elevation of Keap‐1 and restored Nrf2‐driven antioxidant defense. Several studies reported that CP induces toxicity by downregulating the Nrf2 signal [56, 57, 58, 59, 60]. In contrast, treatment with OCA and its combination with EDA significantly restored Keap‐1, Nrf2, GCLC, SOD3, and HO‐1 mRNA expression. EDA antioxidant activity has been attributed to Nrf2 activation in chlorpyrifos‐induced neural toxicity [61]. Similarly, OCA has been reported to modulate Nrf2‐related signaling in rodent models of liver injury [62]. These findings support the involvement of the Nrf2/HO‐1 pathway in mitigating cisplatin‐induced hepatotoxicity and the therapeutic potential of OCA and its combination with EDA in enhancing antioxidant defense.

TNF‐α and interleukins are pivotal in modulating the intricate relationship between inflammatory, oxidative stress, and apoptotic pathways [63, 64]. TNF‐α amplifies hepatic tissue inflammation by attracting immune cells through chemotaxis and activating other cytokines [65]. Interleukins regulate immune cell proliferation and migration, as well as pro‐inflammatory and anti‐inflammatory properties, during the inflammatory response [66]. Injection of CP elicits a robust inflammatory response in liver tissues, as evidenced by increased TNF‐α, IL‐1β, and IL‐6 levels. In the same context, it upregulated NF‐κB expression. These align with several studies that reported inflammation's key role in CP‐associated toxicities [67, 68, 69, 70]. Conversely, OCA and its combination with EDA exhibited notable hepatoprotective effects in rats against CP‐induced liver injury, evidenced by decreased TNF‐α, IL‐1β, and IL‐6 levels, accompanied by reduced NF‐κB expression. Similar results were reported when EDA was used in endotoxin‐induced liver injury [71] and OCA in a cirrhotic model [72]. Our findings suggest that treatment with OCA and EDA attenuates this inflammatory cascade through modulation of distinct but interconnected pathways: OCA treatment was associated with reduced NF‐κB‐related inflammatory signaling, which may be partly related to FXR‐associated mechanisms, while EDA may contribute by reducing oxidative stress linked to inflammation. The combination therapy produced a more pronounced anti‐inflammatory effect.

Our findings suggest that the modulation of the AKT/GSK‐3β pathway plays a critical role in the observed hepatoprotective effects. Activation of GSK‐3β has been reported to promote apoptosis by triggering mitochondrial dysfunction, facilitating cytochrome c release, and activating pathways associated with death receptors [73, 74]. In contrast, AKT is a critical regulator of cell survival. It is known for its anti‐apoptotic role through phosphorylation, suppressing pro‐apoptotic proteins, such as GSK‐3β, while boosting anti‐apoptotic factors, such as Bcl‐2 [75]. Therefore, activation of AKT inhibits GSK‐3β and, consequently, reduces mitochondrial apoptosis and caspase cascade activation [73]. Our investigation demonstrated that CP significantly increased the protein expression of GSK‐3β and p‐GSK‐3β, as well as downregulated the protein expression of p‐AKT. Our study shows that CP reduces AKT phosphorylation in liver tissue, indicating suppression of a critical prosurvival pathway. Decreased AKT activity reduces inhibition of apoptotic effectors such as Bad and Forkhead, leading to hepatocyte death, mitochondrial dysfunction, and liver damage. Notably, oxidative stress and disrupted survival pathways like AKT signaling induce apoptosis, a main cause of liver injury. Thus, reduced AKT phosphorylation underscores cisplatin's role in impairing hepatocyte survival and promoting apoptosis [76]. On the other hand, OCA and its combination with EDA counterbalanced these results, decreased the protein expression of p‐GSK‐3β, and upregulated the protein expression of p‐AKT. Results comply with earlier findings [77]. Herein, CP increased GSK‐3β phosphorylation and inhibited AKT, activating apoptosis. GSK‐3β influences inflammation, metabolism, ER stress, mitochondrial apoptosis, and tissue damage, supporting caspase activation and cell death, linking it to chemotherapy toxicity. Evidence indicates that GSK3β regulates autophagy and apoptosis during tissue injury. Liu et al. found that reducing GSK3β enhances autophagy and protects against CP toxicity by decreasing hair cell loss and apoptosis. This aligns with our observation of increased GSK3β phosphorylation in liver tissues, indicating its role in signaling. GSK‐3β downregulation's protective effect suggests that its activation contributes to cytotoxicity, mitochondrial damage, and apoptosis, which are linked to CP hepatotoxicity [78]. Side by side, CP upregulated GSK‐3β in rats’ testicular injury, associated with oxidative stress, inflammation, and ferroptosis. Vitis vinifera extract reduced GSK3β activity, increased antioxidant levels, and reduced inflammatory and ferroptotic markers, indicating GSK3β‘s protective role. These findings confirm cisplatin toxicity involves GSK3β activation, and targeting this pathway could reduce liver injury [79].

Our investigation demonstrated that CP significantly increased total GSK‐3β and p‐GSK‐3β protein expression while downregulating p‐AKT expression. In contrast, OCA, EDA, and particularly their combination restored p‐AKT signaling and reduced total GSK‐3β abundance. The concomitant reduction in p‐GSK‐3β should be interpreted alongside the decline in total GSK‐3β protein, suggesting an overall attenuation of GSK‐3β‐associated pro‐death signaling rather than defining a specific post‐translational regulatory event. One possible explanation is that alleviation of cisplatin‐induced oxidative stress and inflammation may, in turn, suppress GSK‐3β expression and/or enhance its turnover; however, these mechanisms were not directly examined in the current study and require further investigation.

It is noteworthy that the Keap1/Nrf2/ARE, TNF‐α/NF‐κB, and AKT/GSK‐3β pathways appear to be interconnected in the liver injury response to CP. Oxidative stress may contribute to inflammatory activation and dysregulation of AKT/GSK‐3β signaling, whereas restoring antioxidant defenses may help attenuate downstream inflammatory and injury‐associated responses. Within this framework, OCA and EDA appear to confer protection by coordinating modulation of redox, inflammatory, and stress/survival signaling pathways. However, direct pathway hierarchy and causal interdependence were not specifically established in the present study.

## 5 Conclusions

In conclusion, OCA and EDA, particularly in combination, attenuated CP‐induced hepatotoxicity by coordinating modulation of antioxidant, anti‐inflammatory, and AKT/GSK‐3β‐associated pro‐survival/pro‐death signaling pathways. The findings support attenuation of oxidative‐inflammatory injury together with restoration of hepatocellular survival‐related signaling; however, the precise downstream contribution of AKT/GSK‐3β to apoptotic cell death was not directly established in the present study. These findings support the potential of OCA, particularly in combination with EDA, for further preclinical evaluation as a protective strategy against CP‐induced hepatotoxicity.

## Author Contributions

**Fares E M Ali:** conceptualization, methodology, formal analysis, writing – original draft, writing – review and editing. **Adel G Bakr:** conceptualization, formal analysis, writing – review and editing. **Abdel‐Gawad S Shalkami:** methodology, data curation, writing – review and editing. **Ehab A M El‐Shoura:** conceptualization, methodology, writing – review and editing. **Lamiaa Khalaf Ahmed:** data curation, resources, writing – review and editing. **Ali F Abd‐Elsalam:** data curation, resources, writing – review and editing. **Ahmed A N Ahmed:** data curation, resources, writing – review and editing. **Emad H M Hassanein:** conceptualization, formal analysis, data curation, writing – original draft, writing – review and editing.

## Funding

The authors have nothing to report.

## Ethics Statement

The present animal procedure was approved by the Faculty of Pharmacy ethical committee at Al‐Azhar University (Approval number: ZA‐AS/PH/6/C/2022) and followed the ARRIVE Guidelines for reporting in vivo experiments.

## Consent

The authors have nothing to report.

## Conflicts of Interest

The authors declare no conflicts of interest.

## Supporting information


Supporting File

## Data Availability

The data that support the findings of this study are available from the corresponding author upon reasonable request.
