# Supplementary material for: Obeticholic Acid and Edaravone Protect Against Cisplatin‐Induced Hepatotoxicity Through Modulation of Keap1/Nrf2/ARE, TNF‐α/NF‐κB, and AKT/GSK‐3β Pathways
Source: J Biochem Mol Toxicol. 2026 Sep 3;40(9):e71101. doi: 10.1002/jbt.71101 (PMC13539285; doi:10.1002/jbt.71101)
Supplement: Supplementary file 1 [file JBT-40-e71101-s001.pdf]

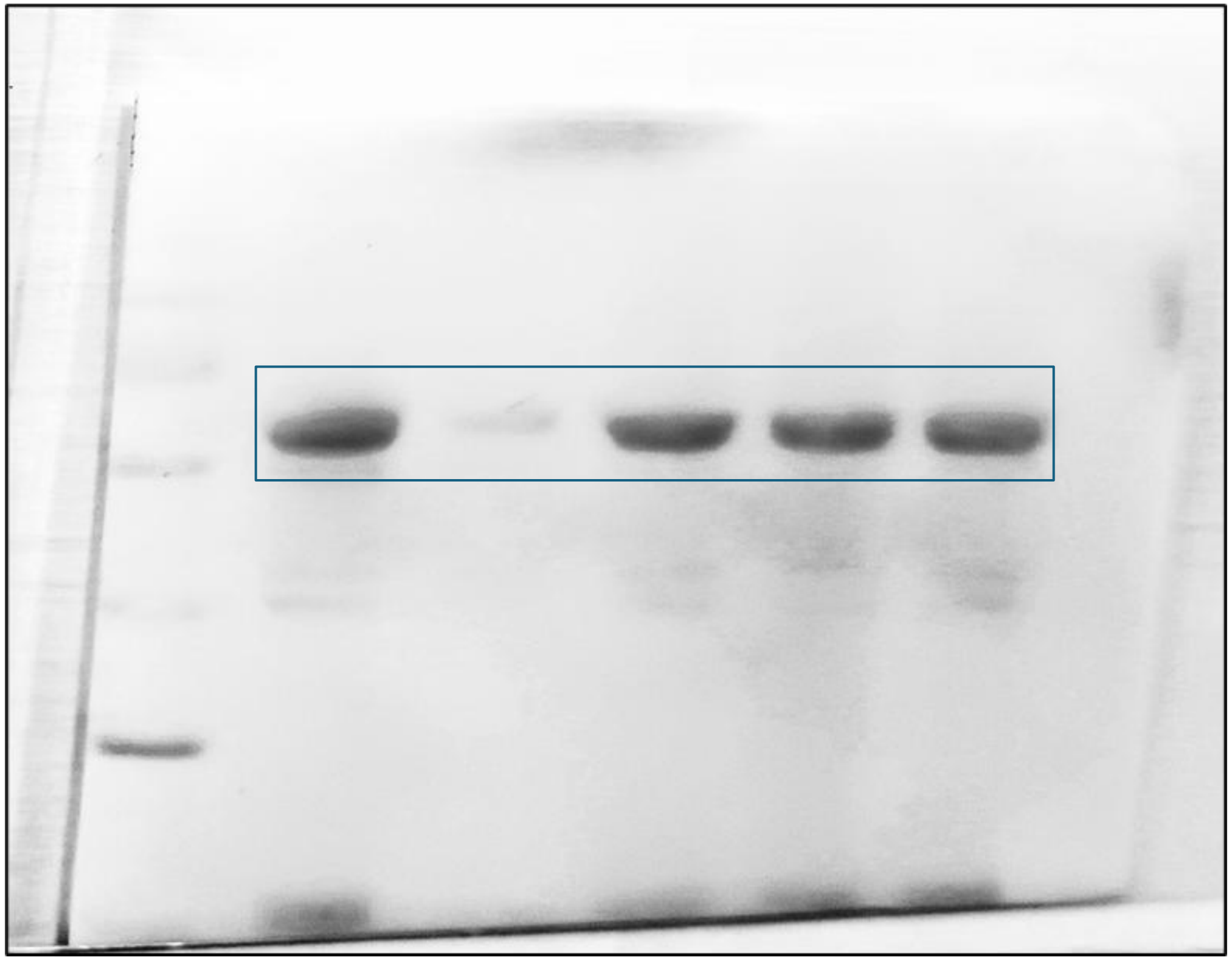

65 KDa

p-AKT

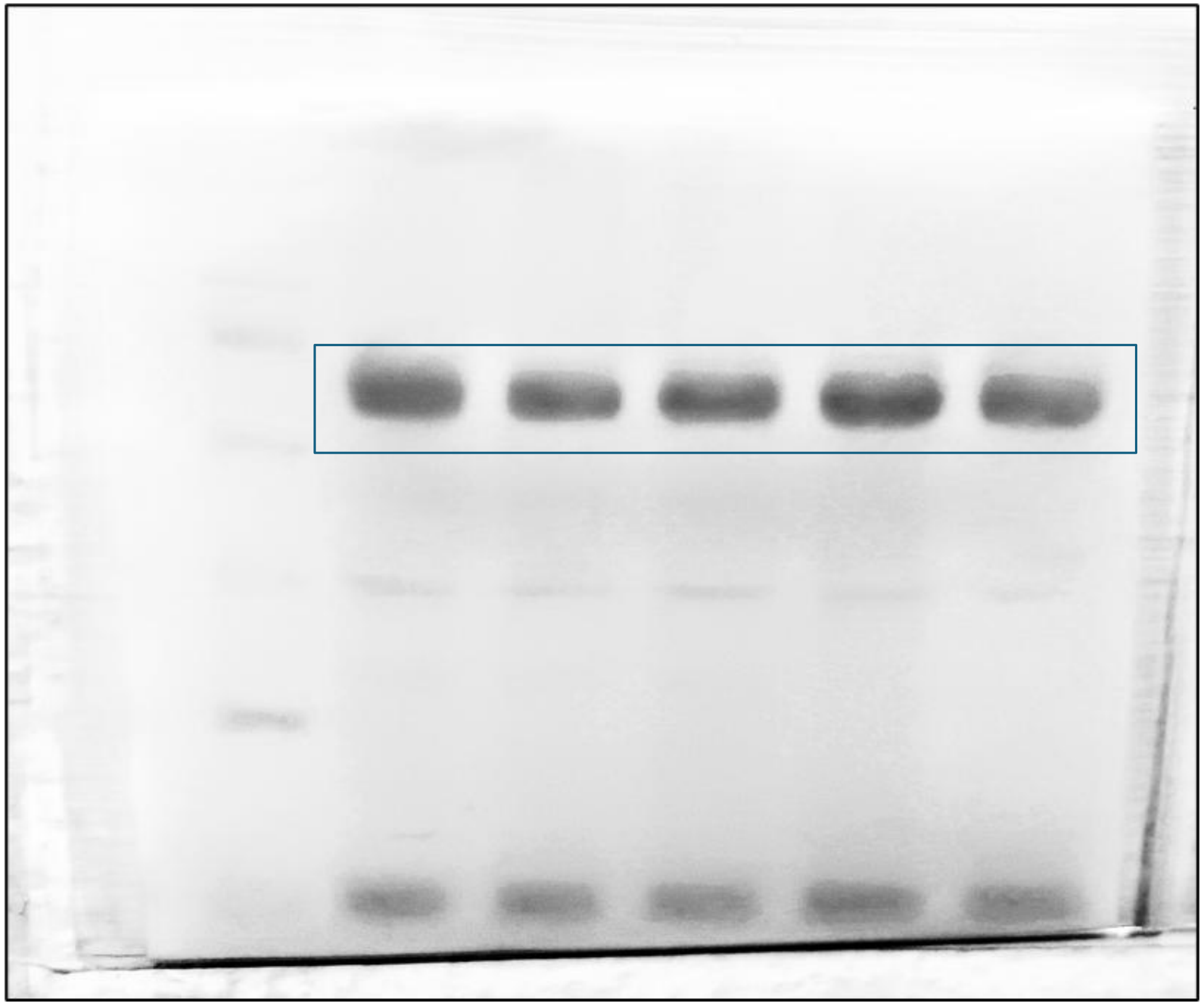

AKT

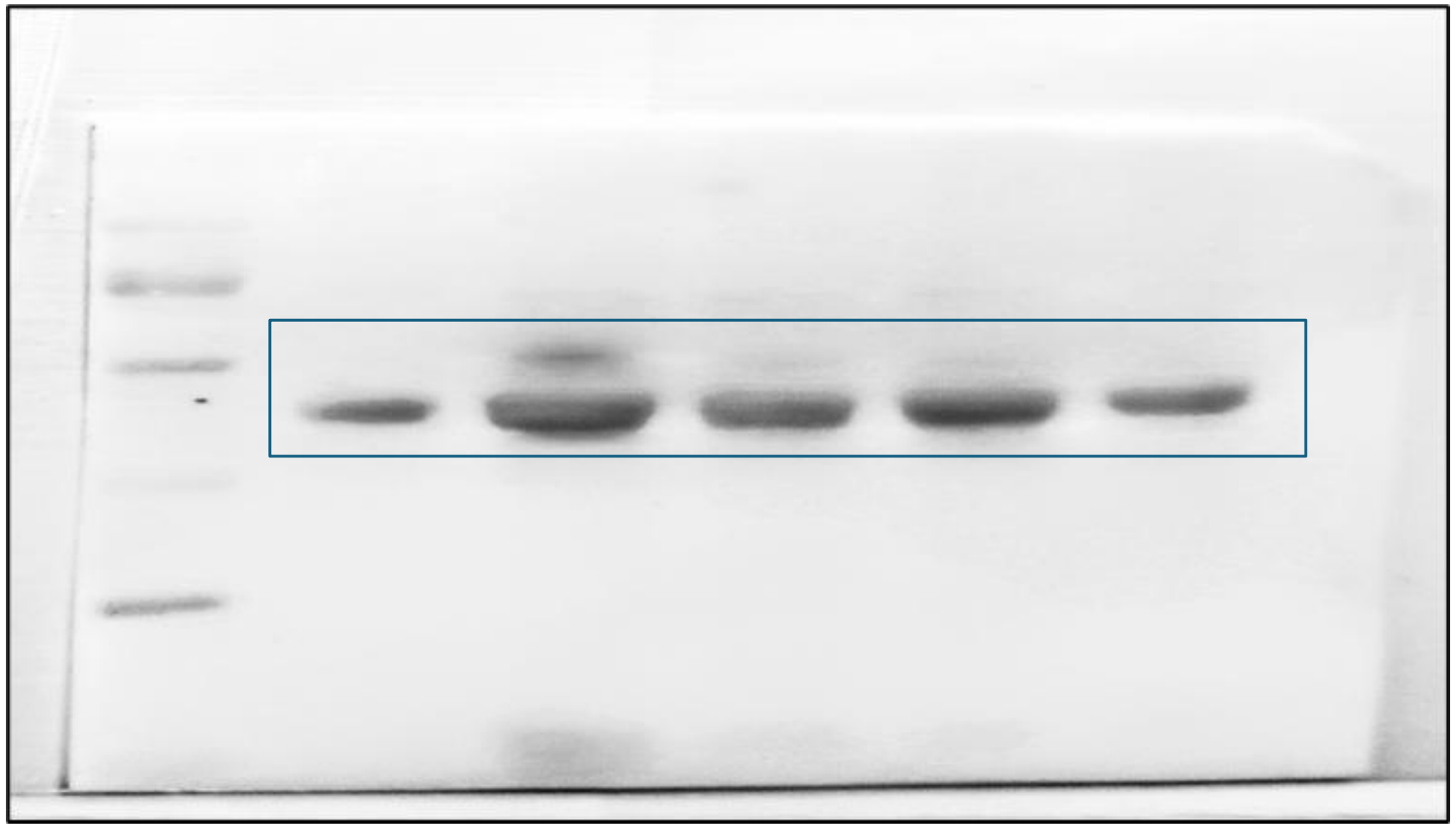

47 KDa

p-GSK-3β

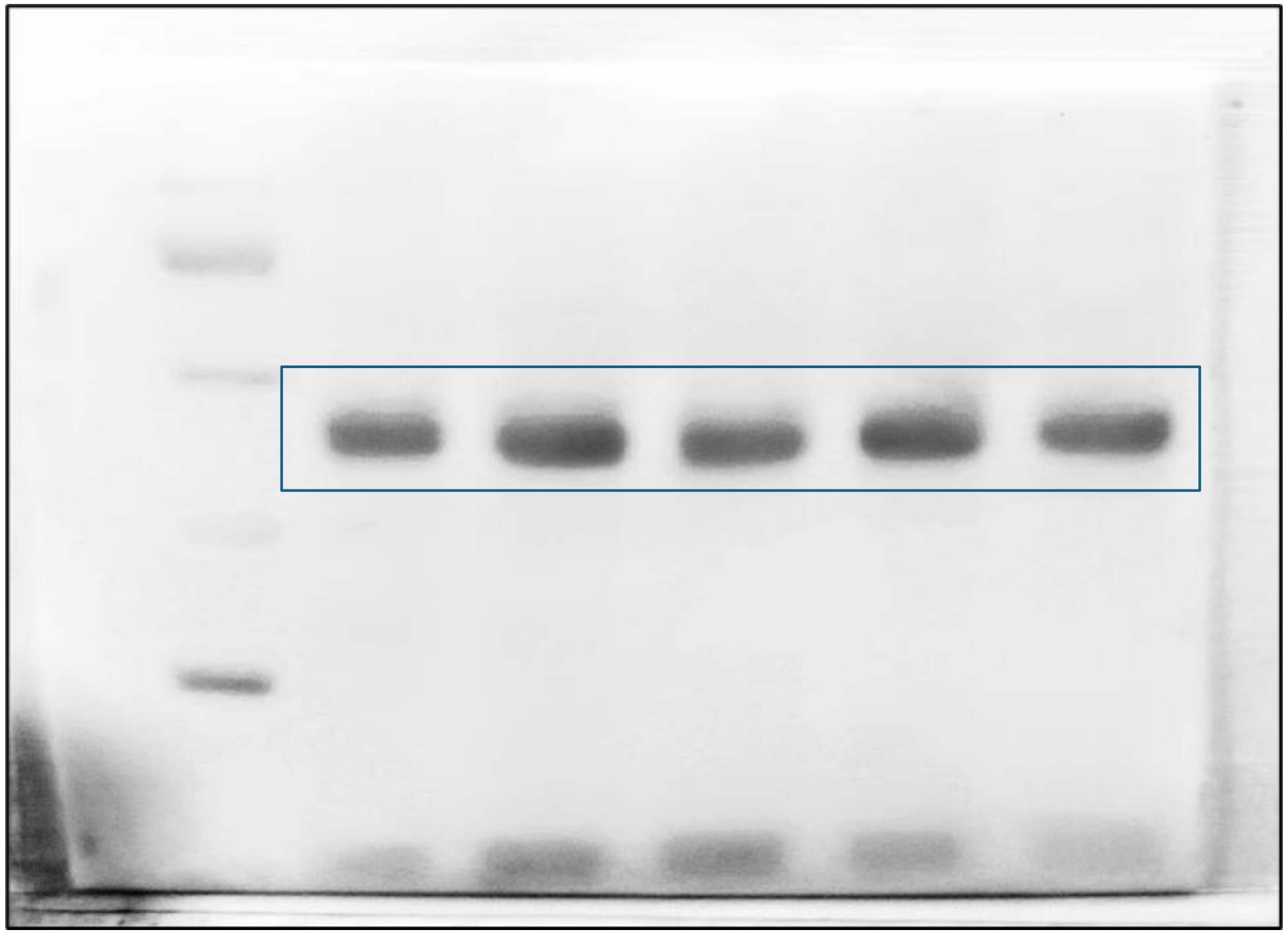

GSK-3β

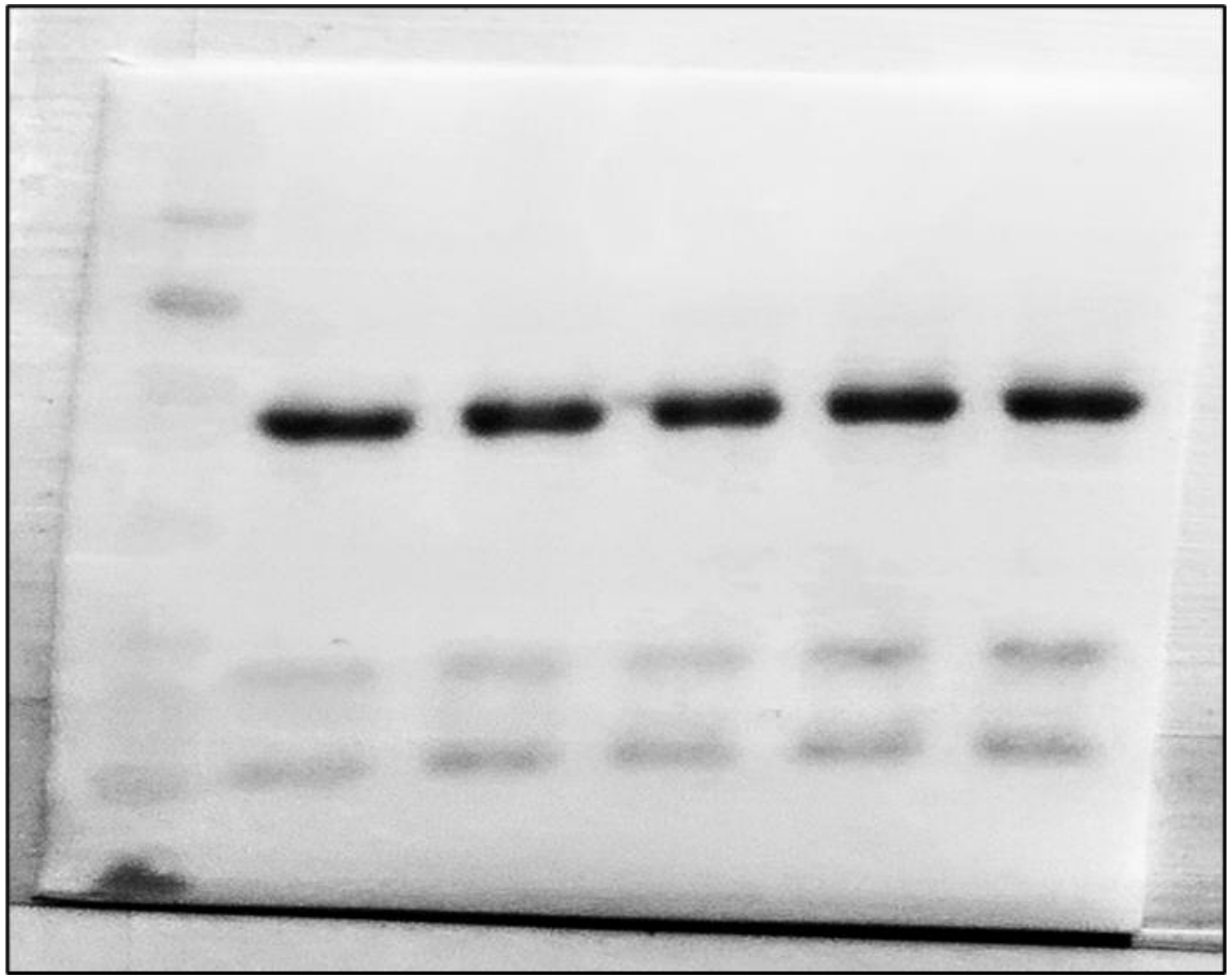

43 KDa

β-actin
